# Clinical significance of *FBXO17* gene expression in high-grade glioma

**DOI:** 10.1186/s12885-018-4680-3

**Published:** 2018-07-31

**Authors:** Di Du, Jian Yuan, Wencai Ma, Jing Ning, John N. Weinstein, Xianrui Yuan, Greg N. Fuller, Yuexin Liu

**Affiliations:** 1 Department of Bioinformatics and Computational Biology, The University of Texas MD Anderson Cancer Center, Houston, TX USA; 2 Department of Neurosurgery, Xiangya Hospital, Central South University, Changsha, Hunan China; 3 Department of Biostatistics, The University of Texas MD Anderson Cancer Center, Houston, Texas USA; 4 Department of Pathology, The University of Texas MD Anderson Cancer Center, Houston, TX USA

**Keywords:** *FBXO17*, High-grade glioma, Gene expression, Epigenetics, Prognosis

## Abstract

**Background:**

High-grade gliomas (HGGs) exhibit marked heterogeneity in clinical behavior. The purpose of this study was to identify a novel biomarker that predicts patient outcome, which is helpful in HGG patient management.

**Methods:**

We analyzed gene expression profiles of 833 HGG cases, representing the largest patient population ever reported. Using the data set from the Cancer Genome Atlas (TCGA) and random partitioning approach, we performed Cox proportional hazards model analysis to identify novel prognostic mRNAs in HGG. The predictive capability was further assessed via multivariate analysis and validated in 4 additional data sets. The Kaplan-Meier method was used to evaluate survival difference between dichotomic groups of patients. Correlation of gene expression and DNA methylation was evaluated via Student’s *t*-test.

**Results:**

Patients with elevated *FBXO17* expression had a significantly shorter overall survival (OS) (*P* = 0.0011). After adjustment by *IDH1* mutation, sex, and patient age, *FBXO17* gene expression was significantly associated with OS (HR = 1.29, 95% CI =1.04–1.59, *P* = 0.018). In addition, *FBXO17* expression can significantly distinguish patients by OS not only among patients who received temozolomide chemotherapy (HR 1.35, 95% CI =1.12–1.64, *P* = 0.002) but also among those who did not (HR = 1.48, 95% CI =1.20–1.82, *P* < 0.0001). The significant association of *FBXO17* gene expression with OS was further validated in four external data sets. We further found that *FBXO17* endogenous expression is significantly contributable from its promoter methylation.

**Conclusion:**

Epigenetically modulated *FBXO17* has a potential as a stratification factor for clinical decision-making in HGG.

**Electronic supplementary material:**

The online version of this article (10.1186/s12885-018-4680-3) contains supplementary material, which is available to authorized users.

## Background

High-grade gliomas (HGGs) are brain tumors associated with high morbidity and mortality [1]. HGGs are classified as either grade III or grade IV (referred to as glioblastoma multiforme, GBM) on the basis of histopathologic features established by the World Health Organization [2]. These tumors demonstrate marked heterogeneity in clinical behavior, with survival durations ranging from less than 1 month to a few years. Accumulating evidence has shown existence of clinically relevant distinct molecular subtypes [3, 4], and molecular predictors of prognosis [5, 6] in HGG. The methylation status of the O-6-methylguanine-DNA methyltransferase (*MGMT*) gene promoter has been shown to be a promising biomarker for predicting GBM response to adjuvant temozolomide (TMZ) chemotherapy [7, 8]. Recently, several studies have used microarray-based gene expression profiling to identify gene signatures capable of predicting clinical outcome of HGG [5, 9–12] but with a limited overlap [5, 11, 13]. To overcome this lack of reproducibility, the best approach is analysis of multiple data sets that have different sample size and patient characteristics. Such analysis applied to microarray data has been shown to be a powerful tool for identifying robust biomarkers [14], however, it is not applicable in most studies. To mimic a large number of data sets for biomarker identification, in this study we developed a random-partitioning methodology and used it in a training set with a large patient population to identify a novel biomarker, *FBXO17* (encoding F-box protein 17), that predicts patient outcome in HGG. We then validated association of *FBXO17* gene expression with patient survival in four additional external data sets, and further investigated the contribution of DNA promoter methylation to *FBXO17* endogenous expression. To the best of our knowledge, we, for the first time, developed this approach and applied it to identify a robust biomarker that could predict patient prognosis in HGG.

## Methods

### Patient samples

The patient clinical annotation and gene expression data used in this study were obtained from publicly available databases. All cases were either grade III or grade IV brain tumors. The databases were chosen on the basis of two criteria: (i) the data sets had both gene expression and survival data; (ii) the number of patients with available data was more than 50. Cases lacking gene expression or survival data were not used for analysis.

The training set consisting of 424 cases was obtained from TCGA [15] and is now available at the TCGA data portal (https://portal.gdc.cancer.gov/). We further obtained a data set of 247 HGG cases from the National Cancer Institute (NCI) Rembrandt (Repository for Molecular Brain Neoplasia Data) database, called ‘Rembrandt Set 2’ in this study. Rembrandt Set 1 (*n* = 183), a subset of Rembrandt Set 2, consisted of the grade IV cases alone. Two additional data sets were obtained from Gene Expression Omnibus (GEO), GSE4271 (*n* = 77) [3] and GSE4412 (*n* = 85) [9]. (A total of 100 samples in GSE4271 had gene expression data, but only 77 had survival data.) Clinical annotation consisted of patient age, gender, survival time, vital status, and grade. TMZ chemotherapy status, *IDH1* (isocitrate dehydrogenase 1) mutation, and *MGMT* status were available only in the TCGA data set.

### Gene expression and DNA methylation analysis

Gene expression data from TCGA were generated using three different platforms (Affymetrix U133, Affymetrix Exon ST, and Agilent 44 K), and a unified expression data set was created and normalized as previously reported [16]. Gene expression data from the three other databases were generated using Affymetrix U133 and were median centered and then log2 transformed. Similar to gene expression, DNA promoter methylation data were also downloaded from TCGA. Pearson correlation of gene expression and DNA methylation was performed and evaluated via Student’s *t*-test.

### Prognostic factor identification

Prognostic markers significantly associated with patient overall survival (OS) were identified in the TCGA training set via a univariate Cox proportional hazards regression model. To compensate for the impact of clinical heterogeneity and sample size on marker identification, we randomly partitioned the training set (*n* = 424) over 500 times, where each partition included a different number of patients (range from 50 to 424) and thus different clinical characteristics [17]. For each partition (here corresponding to a different training set), we applied the Cox proportional hazards model to correlate gene expression with patient survival. As expected, the calculated Cox coefficients and statistical tests varied by partition. The average values of the coefficients and the corresponding *P*-value were calculated from the 500 partitions.

### Statistical analysis

Statistical analysis was performed using Matlab 8.4 (Mathworks, Natick, MA), GraphPad Prism 5.0 (GraphPad Software Inc., La Jolla, CA), or SPSS 18.0 (SPSS Inc., Chicago, IL) as needed. Two different methods were utilized to examine the association of *FBXO17* expression with patient survival. In the Kaplan-Meier survival analysis, patients were dichotomized into either *FBXO17* low group or *FBXO17* high group on the basis of *FBXO17* gene expression levels, and the survival difference between these two groups was assessed with a log-rank test. Univariate and multivariate Cox proportional hazards regression models treated *FBXO17* expression as a continuous variable and used Wald’s test to assess statistical differences in survival. In the multivariate model, *IDH1* mutational status, age, and gender were treated as categorical variables. The Pearson correlation analysis was used to correlate *FBXO17* mRNA expression with promoter DNA methylation with Student’s *t*-test. The median age difference among the different data sets was assessed via Kruskal-Wallis test. The *FBXO17* mRNA expression difference between patients who received TMZ chemotherapy and those who did not was evaluated via Mann Whitney test. All statistical tests were two-sided, and a *P*-value < 0.05 was considered statistically significant.

## Results

### Clinicopathologic findings

Clinical characteristics of patients included in the cohort and in the four validation cohorts, comprising a total of 833 HGG cases, are described in Table 1. Both the TCGA and Rembrandt Set 1 data sets contained GBM exclusively. Rembrandt Set 2, GSE4271, and GSE4412 contained both grade III and IV tumors, with percentages of grade III tumors ranging from 25 to 31% (*P* = 0.70, chi-square test). The median ages were significantly younger in GSE4271 and GSE4412 than in the other data sets. (*P* < 0.0001, Additional file 1: Figure S1). The five data sets differed significantly in patient OS (*P* < 0.0001, Additional file 2: Figure S2); GSE4271 had much longer median OS. Different from the other four data sets, GSE4412 had a significantly larger number of female patients (*P* < 0.0001, Fisher’s exact test).

**Table 1:** Clinicopathologic characteristics of the patients in the five HGG data sets

	TCGA	Rembrandt 1^b^	Rembrandt 2^c^	GSE4271	GSE4412
No. of patients^a^	424	183	247	77	85
Age, median (range)	58.9 (10.9–89.3)	50 (20–80)	50 (15–80)	45 (22–82)	42 (18–82)
Sex					
Female	159 (37.5)	47 (36.4)	67 (38.5)	25 (32.5)	53 (62.4)
Male	265 (62.5)	82 (63.6)	107 (61.5)	52 (67.5)	32(37.6)
NA	0	54	73	0	0
Grade					
III	0 (0)	0 (0)	64 (25.9)	21 (27.3)	26 (30.6)
IV (GBM)	424 (100)	183 (100)	183 (74.1)	56 (72.7)	59 (69.4)
Vital Status					
Alive	75 (17.7)	10 (5.5)	21 (8.5)	15 (19.5)	26 (30.6)
Dead	349 (82.3)	173 (94.5)	226 (91.5)	62 (80.5)	59 (69.4)
Median OS in months	14.2	15	16	23.8	13.9

Values are reported as No. (%) unless otherwise indicated. Missing values are excluded from the percentage calculation. *NA* not applicable

^a^Samples with both OS (vital status) and gene expression data

^b^GBM sample only from Rembrandt database

^c^GBM and Grade III astrocytoma/oligodendroglioma from Rembrandt database

### Identification of *FBXO17* as a novel prognostic factor in the TCGA GBM dataset

**Fig. 1:**
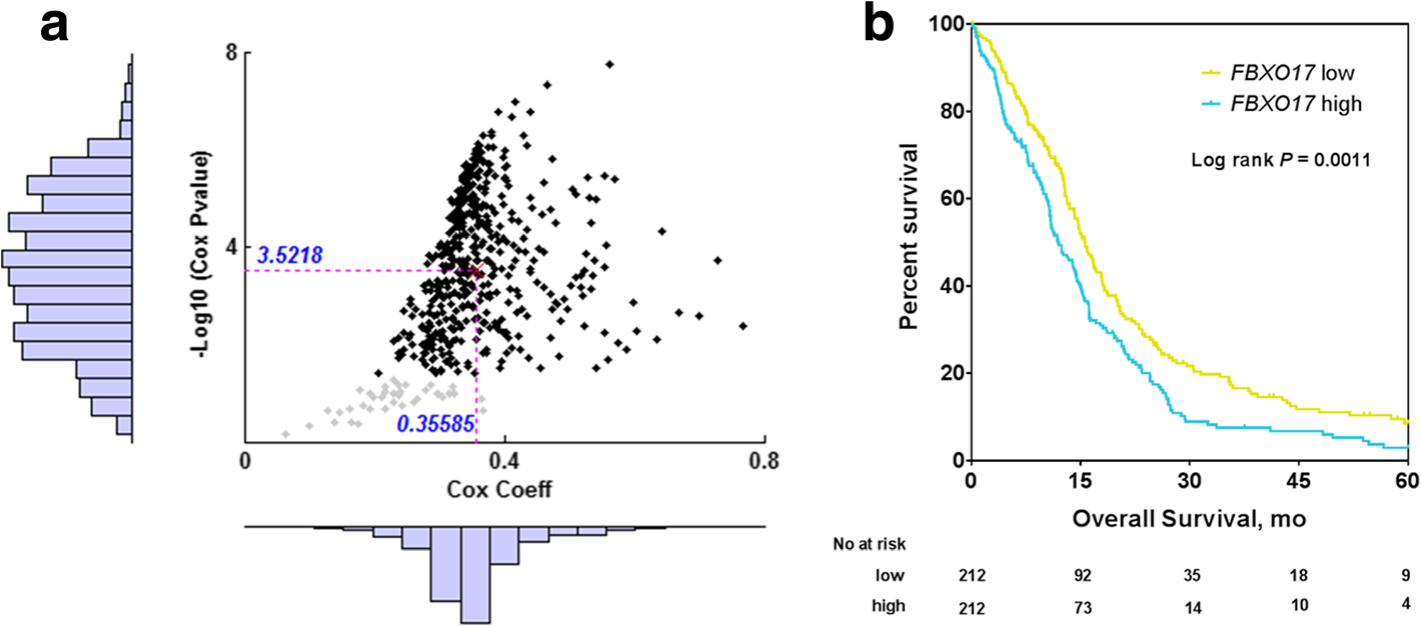
Identification of *FBXO17* as a novel prognostic factor in the TCGA GBM data set. **a** Association of *FBXO17* expression with patient OS via Cox proportional hazards model for 500 partitions of the TCGA GBM cohort. The histograms indicate distribution of the Cox coefficients and the corresponding statistical tests. The median values of the *P*-values and Cox coefficients among the 500 partitions are also shown. **b** Kaplan-Meier OS curves for the TCGA GBM set after classifying patients into two groups based on the median of *FBXO17* gene expression

We performed univariate Cox proportional hazards regression analysis to correlate genome-wide gene expression profiling with patient survival in the TCGA cohort and found that *FBXO17* was among the top 10 most significantly correlated genes and hasn’t been well studied yet in literatures (Additional file 3: Table S1). Moreover, *FBXO17* expression was significantly correlated with patient OS (*P* < 0.05) in 463 (approximately 92.6%) out of 500 random partitions (see Methods for details) and the median *P*-value of all 500 statistical tests was approximately 3 × 10^− 4^ (Fig. 1a). The positive Cox coefficients indicated that low *FBXO17* expression was significantly associated with better survival than was high *FBXO17* expression. As compared to the Cox coefficients, *P* values are widely distributed, suggesting that partitions have much impact on statistical tests.

Next, we dichotomized all the patients into two groups based on the median of *FBXO17* gene expression, low and high. Kaplan-Meier survival analysis showed that these two groups differed significantly in OS duration; the median survival of patients with a high level of *FBXO17* was about 11.9 months, significantly shorter than that of patients with a low level of *FBXO17* (15.6 months, log rank *P* = 0.0011, Fig. 1b).

### Validation of *FBXO17* in multiple external data sets

**Fig. 2:**
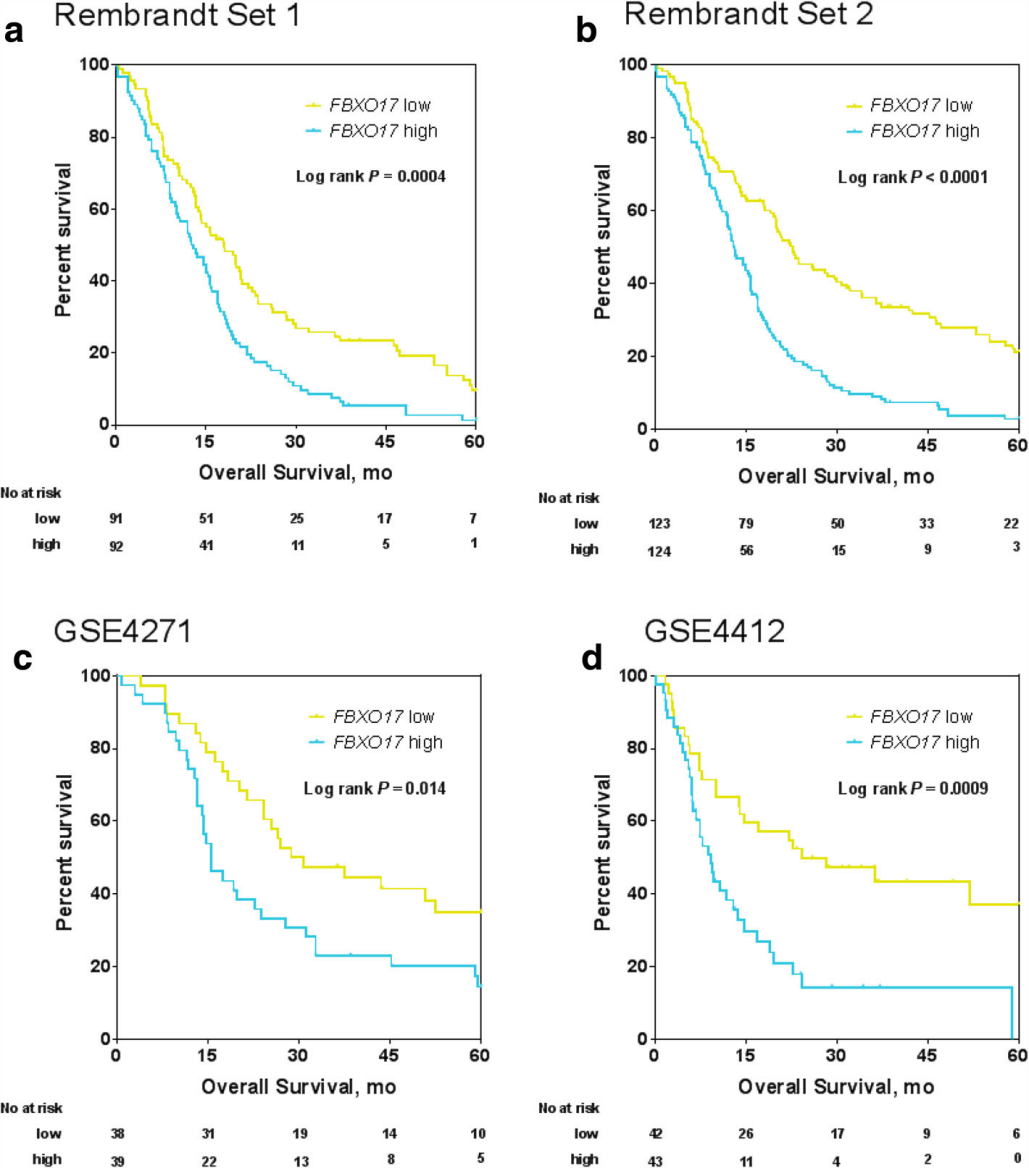
Validation of FBXO17 in multiple external data sets. Kaplan-Meier OS curves after classifying patients into two groups based on *FBXO17* gene expression in (**a**) Rembrandt Set 1 (GBM only), (**b**) Rembrandt Set 2 (GBM + grade III gliomas), (**c**) GSE4271, and (**d**) GSE4412

To evaluate the robustness and scalability of *FBXO17* as a prognostic predictor, we sought to validate correlation of *FBXO17* mRNA expression with OS in the four publically available data sets. In each of the validation cohorts, patients were dichotomized into either *FBXO17* high or low group based on the median of the *FBXO17* gene expression. Specifically, in Rembrandt Set 1, the median survival for those in the *FBXO17*-high group was ~ 12.8 months, versus ~ 18.0 months for those in the *FBXO17*-low group, with statistical significance of *P* = 0.0002 (Fig. 2a). The survival difference of the high versus low groups was statistically significant across the other three data sets, specifically in Rembrandt Set 2 (*P* < 0.0001, Fig. 2b), in the GSE4271 set (*P* = 0.014, Fig. 2c) and in the GSE4412 set (*P* = 0.0009, Fig. 2d). We repeated this survival analysis of Grade IV glioma patients alone in both GSE4271 and GSE4412 data sets (Additional file 4: Figure S3). Patients with higher *FBXO17* gene expression exhibited a trend toward a worse survival than those with lower *FBXO17* expression though the statistical significance was not reached likely because of smaller size of analyzed samples in this analysis. In all, a high level of *FBXO17* expression was consistently associated with a worse prognosis in HGG.

To test whether this result was independent of *IDH1* mutation, a known prognostic factor in GBM [15], we next incorporated *IDH1* mutation status into survival analysis. It should be mentioned that *IDH1* mutation status is available only in the TCGA cohort, unfortunately not in the other four data sets. Integration of whole-exome sequencing data revealed that total 311 GBM patients had clinical, mutation, and gene expression data, among which 23 cases (~ 7.40%) harbored *IDH1* mutations. Consistent with previous reports [15], patients with *IDH1* mutations had significantly better survival than the wide-type cases (HR = 0.38, 95% CI = 0.21–0.68, *P* = 0.001, Table 2). Next, we applied multivariate analyses using a Cox proportional hazards model with *FBXO17* gene expression, *IDH1* mutation status and other clinicopathologic factors as covariates. After adjustment by *IDH1* mutation, sex, and patient age, *FBXO17* gene expression was significantly associated with shorter survival (HR = 1.29, 95% CI = 1.04–1.59, *P* = 0.018, Table 2). *IDH1* mutation was not significantly associated with survival in this adjusted model, likely because *IDH1* mutation frequency is low in GBM patients (typically less than 10%). Next we included *MGMT* methylation status as a covariate into the multivariate model, the number of cases that were available for analysis (*n* = 210) was significantly reduced due to missing values. In this smaller cohort, none of these three factors including *IDH1* mutation, *MGMT* methylation and *FBXO17* gene expression were significantly correlated with survival (Additional file 5: Table S2). Recently, a G-CIMP phenotype characteristic of a CpG island methylator was identified to be associated with *IDH1* mutation [15]. We found that *FBXO17* expression was significantly lower in the G-CIMP subtype and significantly higher in the Classical subtype (Additional file 6: Figure S4). To further alleviate the impact from *IDH1* mutations, we performed additional analyses while excluding the *IDH1* mutant cases. Even among the *IDH1*-widetype patients alone, *FBXO17* gene expression was significantly associated with shorter survival both in the univariate model (HR = 1.33, 95% CI = 1.08–1.64, *P* = 0.007) and in the adjusted model (HR = 1.26, 95% CI = 1.02–1.56, *P* = 0.034) (Table 2). These data suggested that *FBXO17* expression is an independent predictor of survival in HGG.

**Table 2:** Univariate and Multivariate Models for Overall Survival in Patients with GBM in the Cancer Genome Atlas (TCGA) Cohort^a^

Overall Survival	Univariate analysis		Multivariate analysis^d^	
	HR (95%CI)^c^	*P* Value^e^	HR (95%CI)^c^	*P* Value^e^
*IDH1* mut + wt cases (*n* = 311)^b^				
*FBXO17* expression	1.47 (1.24–1.74)	< 0.0001	1.29 (1.04 --1.59)	0.018
*IDH1* status				
Wild-type (288)	1 [Reference]		1 [Reference]	
Mutation (23)	0.38 (0.21–0.68)	0.001	0.62 (0.31 --1.25)	0.179
Sex				
Female (113)	1 [Reference]		1 [Reference]	
Male (198)	1.11 (0.85–1.45)	0.451	1.18 (0.90 --1.55)	0.216
Age, years				
< 60 (164)^f^	1 [Reference]		1 [Reference]	
≥ 60 (147)	1.74 (1.34 -- 2.26)	< 0.0001	1.56 (1.20 -- 2.03)	0.001
*IDH1* wt cases alone (*n* = 288)				
*FBXO17* expression	1.33 (1.08–1.64)	0.007	1.26 (1.02 --1.56)	0.034
Sex				
Female (106)	1 [Reference]		1 [Reference]	
Male (182)	1.23 (0.94–1.62)	0.138	1.23 (0.94 --1.62)	0.139
Age, years				
< 60 (144)	1 [Reference]		1 [Reference]	
≥ 60 (144)	1.69 (1.29 -- 2.21)	< 0.0001	1.59 (1.21 -- 2.09)	0.001

^a^Included are data from the 311 TCGA GBM patients who had all the following data available such as FBXO17 gene expression, IDH1 mutation, gender, age, survival time, and vital status. The numbers in parentheses denote the numbers of patients in the specified subgroups

^b^wt, wide-type; mut, mutant

^c^HR, hazard ratio; CI, confidence interval

^d^Based on a multivariate Cox proportional hazards model, including all variables in the table

^e^Wald’s test, *P* values

^f^The median age of the common set is 60 years

### *FBXO17* predicts survival regardless of TMZ chemotherapy

**Fig. 3:**
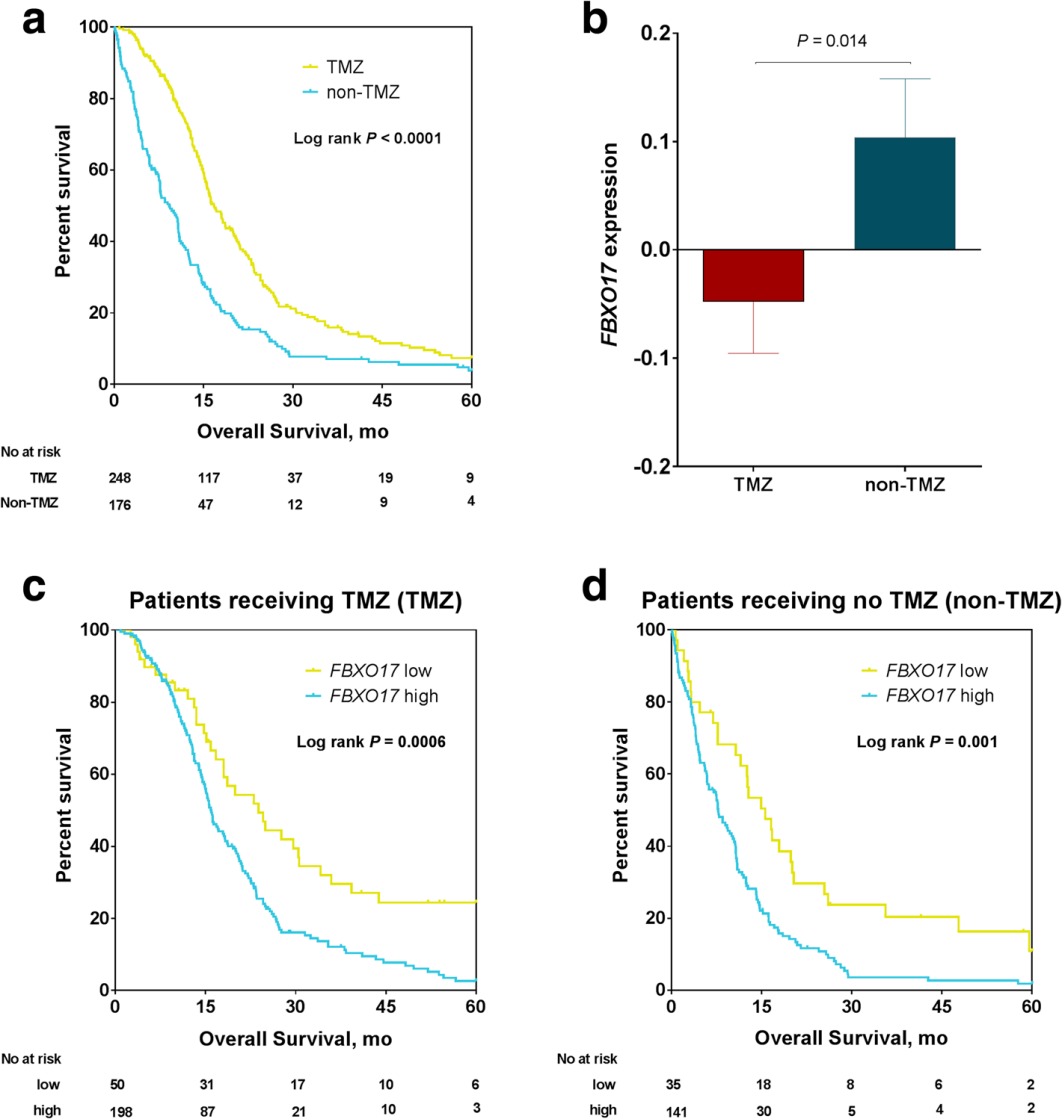
*FBXO17* predicts survival regardless of TMZ chemotherapy. **a** Kaplan-Meier OS curves for patients who received TMZ chemotherapy and patients who did not receive TMZ. **b** Association of *FBXO17* expression with TMZ chemotherapy. **c** Kaplan-Meier OS curves for patients in the TMZ group after classifying patients into two groups based on *FBXO17* gene expression. **d** Kaplan-Meier OS curves for patients in the non-TMZ group after classifying patients into two groups based on *FBXO17* gene expression

Temozolomide (TMZ) is an FDA-approved chemotherapy drug used for the treatment of GBM patient [18]. When analyzing the TCGA GBM data set, we found that patients who received TMZ chemotherapy had significantly longer survival than those who did not (*P* < 0.0001, Fig. 3a). Consistent with its prognostic association as stated above, *FBXO17* expression was significantly lower in the TMZ group than in the non-TMZ group (*P* = 0.014, Mann Whitney test, Fig. 3b). We next interrogated the prognostic utility of *FBXO17* expression with regard to TMZ chemotherapy using two different approaches. Among patients who received TMZ chemotherapy, the Cox proportional hazards model showed *FBXO17* expression was significantly correlated with OS (HR = 1.35, 95% CI = 1.12–1.64, *P* = 0.002,), and the Kaplan-Meier method demonstrated that patients with higher *FBXO17* expression had significantly shorter survival than those with lower *FBXO17* expression (*P* = 0.0006, Fig. 3c). This result is similar to the impact of *MGMT* status on survival in this subset of GBM; patients with methylated *MGMT* had significantly longer survival than those with unmethylated *MGMT* (*P* = 0.018, Additional file 7: Figure S5a), which is consistent with its role in predicting response to TMZ chemotherapy.

Even for those who did not receive TMZ chemotherapy and typically had worse prognosis (Fig. 3a), the Cox model showed that *FBXO17* expression was still significantly associated with patient survival (HR = 1.48, 95% CI = 1.20–1.82, *P* < 0.0001), and the Kaplan-Meier method indicted that the median survival of *FBXO17*-expression low patients was two times longer than that of *FBXO17*-expression high patients (*P* = 0.001, Fig. 3d). Different from *FBXO17* gene expression, *MGMT* status was not correlated with survival in this subset of GBM who didn’t receive TMZ treatment (*P* = 0.965, Additional file 7: Figure S5b). Of note, *FBXO17* expression was not correlated with *MGMT* methylation status (*P* = 0.154, Additional file 8: Figure S6).

### DNA promoter hypermethylation silences *FBXO17* mRNA expression

**Fig. 4:**
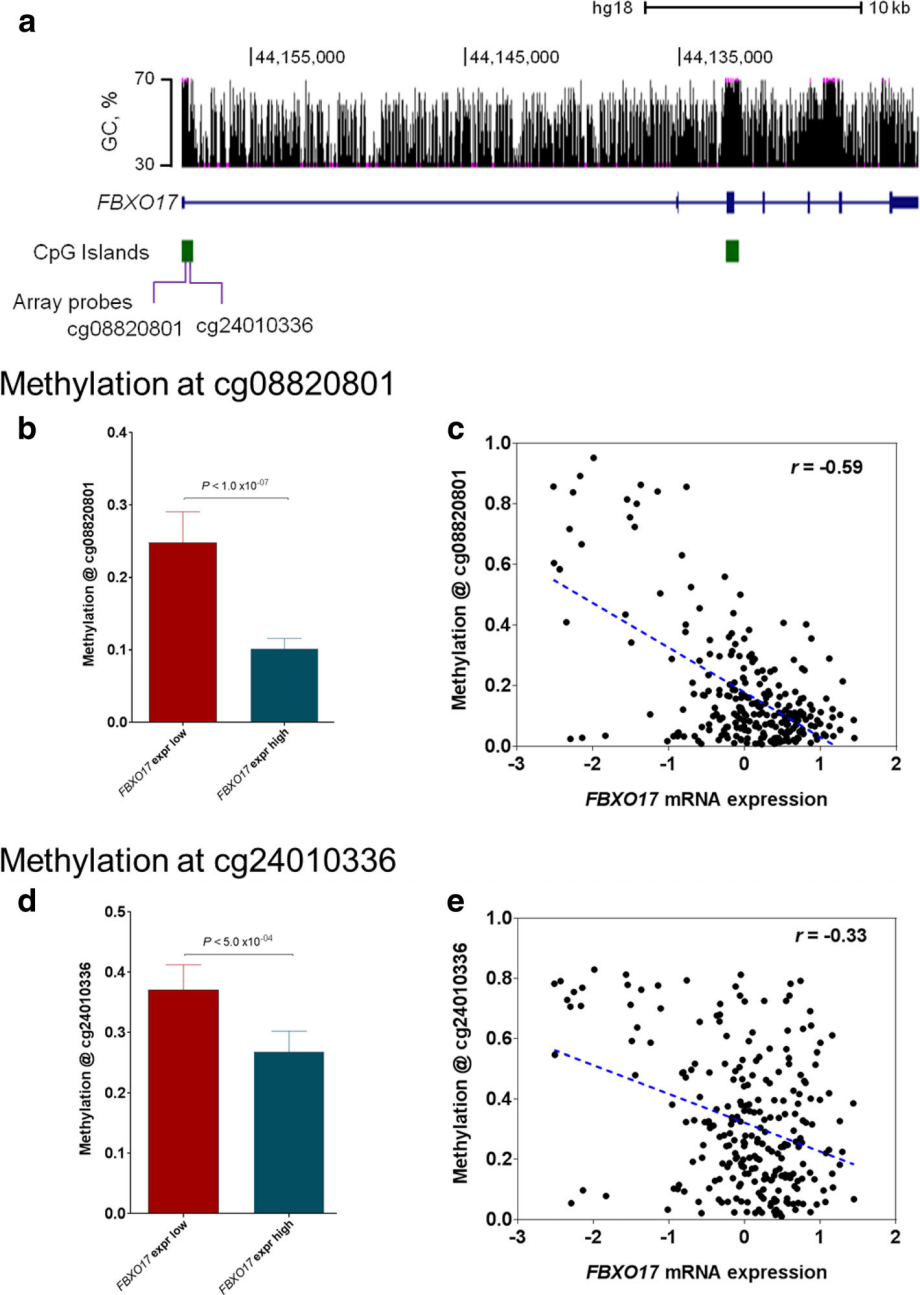
DNA Promoter hypermethylation silences *FBXO17* mRNA expression. **a** GC percentage, CpG islands, genomic loci of *FBXO17* gene, and the two methylation probes annotated to *FBXO17* in DNA methylation microarray are indicated. **b** Comparison of DNA methylation beta values at cg08820801. **c** Correlation of *FBXO17* mRNA expression with promoter DNA methylation at the probe cg08820801. **d** Comparison of DNA methylation beta values at cg24010336. **e** Correlation of *FBXO17* mRNA expression with promoter DNA methylation at cg08820801

*FBXO17,* encoding F-box protein 1, is known to be involved in ubiquitination and glycoprotein catabolism [19, 20] and was reported to be hypomethylated in adenoid cystic carcinoma of the salivary gland [21]. To investigate the genetic determinant of *FBXO17* endogenous expression, we next correlated *FBXO17* gene expression with its DNA methylation [22]. The *FBXO17* gene, located at chromosome 19q13.2, contains a high percentage of GC bases in the form of CpG islands. Two probes, cg08820801 and cg24010336, are present in the CpG island, 559 and 878 base pairs, respectively, away from the transcription start site (Fig. 4a). Integration of TCGA GBM data sets identified that 253 patients had both gene expression and DNA methylation data. The *FBXO17* DNA methylation beta values at cg08820801 were significantly higher in the low-*FBXO17*-expression group (*n* = 126) than in the high-expression group (*n* = 127) (*P* < 1.0 × 10^− 07^, Mann Whitney test, Fig. 4b), and were negatively correlated with *FBXO17* mRNA expression (Pearson correlation coefficient, *r* = − 0.59, *P* = 2.2 × 10^− 25^, Student’s *t*-test, Fig. 4c). Similarly, the *FBXO17* DNA methylation beta values at cg24010336 were significantly higher in the low-*FBXO17*-expression group (*P* < 5.0 × 10^− 04^, Fig. 4d), and were negatively correlated with *FBXO17* mRNA expression (*r* = − 0.33, *P* = 5.1 × 10^− 08^, Fig. 4e). Similar to *FBXO17* gene expression, *FBXO17* promoter methylation was also significantly correlated with patient survival but with an opposite pattern, that is, patients with higher *FBXO17* promoter methylation had significantly better survival than those with lower *FBXO17* promoter methylation (Additional file 9: Figure S7). Taken together, these results collectively suggest that *FBXO17* mRNA endogenous expression is likely attributable to promoter DNA methylation.

## Discussion

In this study of genomic data for a total patient population of 833 HGG cases, we first developed a random partitioning approach to identify *FBXO17* as a novel prognostic factor in the TCGA GBM cohort, and then validated its prognostic utility in 4 external HGG cohorts. Multivariate analysis further demonstrated that *FBXO17* was significantly correlated with survival after adjustment by *IDH1* mutations. In addition, our results showed that *FBXO17* expression can distinguish patients by OS not only for patients who received TMZ treatment, but also for those who didn’t. Integrative analysis showed that *FBXO17* endogenous expression is significantly contributable from promoter methylation. To truly determine its clinical utility, the immunohistochemical analysis of *FBXO17* protein on HGG tumor specimens is required and deserves detailed investigation in the future. The single-gene measurement assay is applicable in clinical practice and the data can be used to evaluate the *FBXO17*’s translational role as a prognostic marker in the clinical arena.

Identification of molecular biomarkers predictive of survival in HGG is important for effectual patient management and represents a crucial step toward individualized treatment. Although several gene signatures have been reported to predict patient outcome in HGG [5, 9–12], only a few genes have been consistently identified [5, 11–13]. Tumor heterogeneity proves to be a primary cause of such small overlap between different studies. To partially account for the heterogeneity issue, a conventional approach is to select markers as those that were identified from a training set and subsequently validated in a test set [23]. Both the training set and test set are usually partitioned from a single sample set. A slightly improved approach to selecting biomarkers is to identify overlapping genes by simultaneously analyzing a few different data sets [5, 11], but this approach requires availability of multiple data sets, which is not applicable in most studies. Nevertheless, the number of data sets used for identifying the markers is still quite limited, far from representing clinical characteristics or genetic background of a particular cancer type.

In contrast to the previous approaches, in this study we randomly partitioned the training set with a large number of patients many times; each partition emulates a training set for biomarker identification. The markers identified to be consistent in the majority of these partitions should be robust, and to a large extent, reflect the tumor heterogeneity. To the best of our knowledge, this is the first time such an approach has been used to identify a robust molecular biomarker that could predict clinical outcome in HGG. Of note, the two conventional approaches mentioned above are actually simplified forms of our current approach.

Compared to genetic biomarkers such as methylation of the *MGMT* promoter and mutations of the *IDH1* gene [24], transcriptome biomarkers, reflecting mRNA levels, are more vulnerable because the expression level of an individual gene is largely dependent on a wide variety of regulatory factors, such as transcription factors, microRNA, and genetic alterations. These complicated regulatory networks make it difficult to identify robust gene expression biomarkers and have contributed, at least in part, to lack of reproducibility in different studies. Following this rationale, a gene expression marker identified to be associated with its own genetic alteration—for instance, mutation, methylation, or copy-number alteration—will likely be less susceptible to impact from other regulatory factors and therefore more robust to tumor heterogeneity. In this study, we found that *FBXO17* mRNA expression was significantly anti-correlated with its promoter DNA methylation. Therefore, we conclude that epigenetically-silenced *FBXO17* is a novel and robust marker predicting patient survival of HGG.

Prognostic biomarkers could provide novel insights into tumor biology and represent potential therapeutic targets. Although *MGMT* is a known predictive marker for patients who receive TMZ chemotherapy due to its involvement in treatment response, no molecular predictor has been reported for those who do not receive TMZ chemotherapy. Our results show that *FBXO17* can predict clinical outcome not only for TMZ-treated patients but also for non-TMZ-treated patients. Moreover, a marker identified to be altered at both the RNA and DNA level is likely a driver [25]. Therefore, *FBXO17* could be a potential target for therapeutic intervention, especially for patients who do not receive TMZ chemotherapy. To fully understand and exploit these results, functional studies are needed. Another limitation of this study is that our findings pertain to *IDH1* mutation and *MGMT* status need to be further validated.

## Conclusions

In conclusion, these results suggest the potential of taking into account *FBXO17* as a stratification factor for HGG patient management, though the prognostic utility needs to be further investigated in a prospective clinical study.

## Additional files

Additional file 1: **Figure S1.** Patient ages in the data sets that are included in this study. (PDF 36 kb)

Additional file 2: **Figure S2.** Survival difference among the data sets that are included in this study. (PDF 57 kb)

Additional file 3: **Table S1.** The top 50 most significantly correlated genes with survival in the TCGA cohort. (DOCX 25 kb)

Additional file 4: **Figure S3.** Correlation of FBXO17 mRNA expression with survival within grade IV glioma patients alone, respectively, (a) in GSE4271 and (b) in GSE4412. The one-third patients with the least FBXO17 expression were categorized into FBXO17 low group and the rest of patients were categorized into FBXO17 high group. (PDF 113 kb)

Additional file 5: **Table S2.** Univariate and Multivariate Models for Overall Survival in Patients with GBM in the TCGA Cohort while including MGMT methylation status as a covariate*. (DOCX 24 kb)

Additional file 6: **Figure S4.** Correlation of FBXO17 mRNA expression with GBM transcriptional subtypes in the TCGA cohort. (PDF 59 kb)

Additional file 7: **Figure S5.** (a) Kaplan-Meier OS curves for patients in the TMZ group after classifying patients into two groups based on MGMT status. (b) Kaplan-Meier OS curves for patients in the non-TMZ group after classifying patients into two groups based on MGMT status. (PDF 46 kb)

Additional file 8: **Figure S6.** Correlation of FBXO17 gene expression with MGMT methylation status in the TCGA cohort. The statistical significance was assessed by Mann-Whitney test. (PDF 65 kb)

Additional file 9: **Figure S7.** Correlation of FBXO17 promoter methylation with patient survival in the TCGA cohort. (PDF 49 kb)

## Abbreviations

FBXO17: F-box protein 17
GBM: Glioblastoma multiforme
GEO: Gene expression omnibus
HGG: High-grade glioma
IDH1: Isocitrate dehydrogenase 1
MGMT: O-6-methylguanine-DNA methyltransferase
OS: Overall survival
Rembrandt: Repository for Molecular Brain Neoplasia Data
TCGA: The Cancer Genome Atlas
TMZ: Temozolomide
