# Supplementary figures and images for: Clinical significance of FBXO17 gene expression in high-grade glioma
Source: BMC Cancer. 2018 Jul 31;18:773. doi: 10.1186/s12885-018-4680-3 (PMC6069786; doi:10.1186/s12885-018-4680-3)

Figure S1

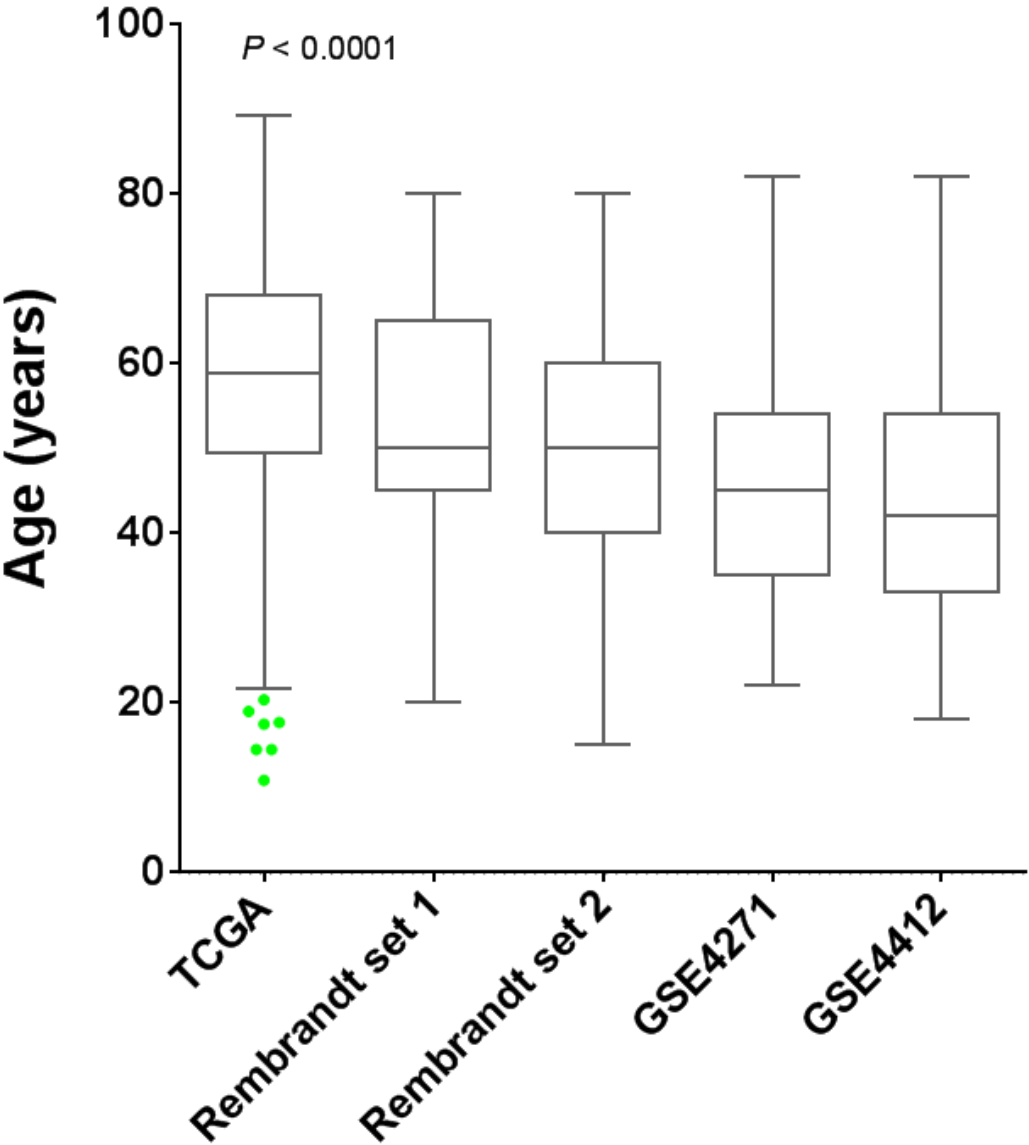

Supplement: Supplementary file 1 — Figure S1. Patient ages in the data sets that are included in this study. (PDF 36 kb) [file 12885_2018_4680_MOESM1_ESM.pdf]

Figure S2

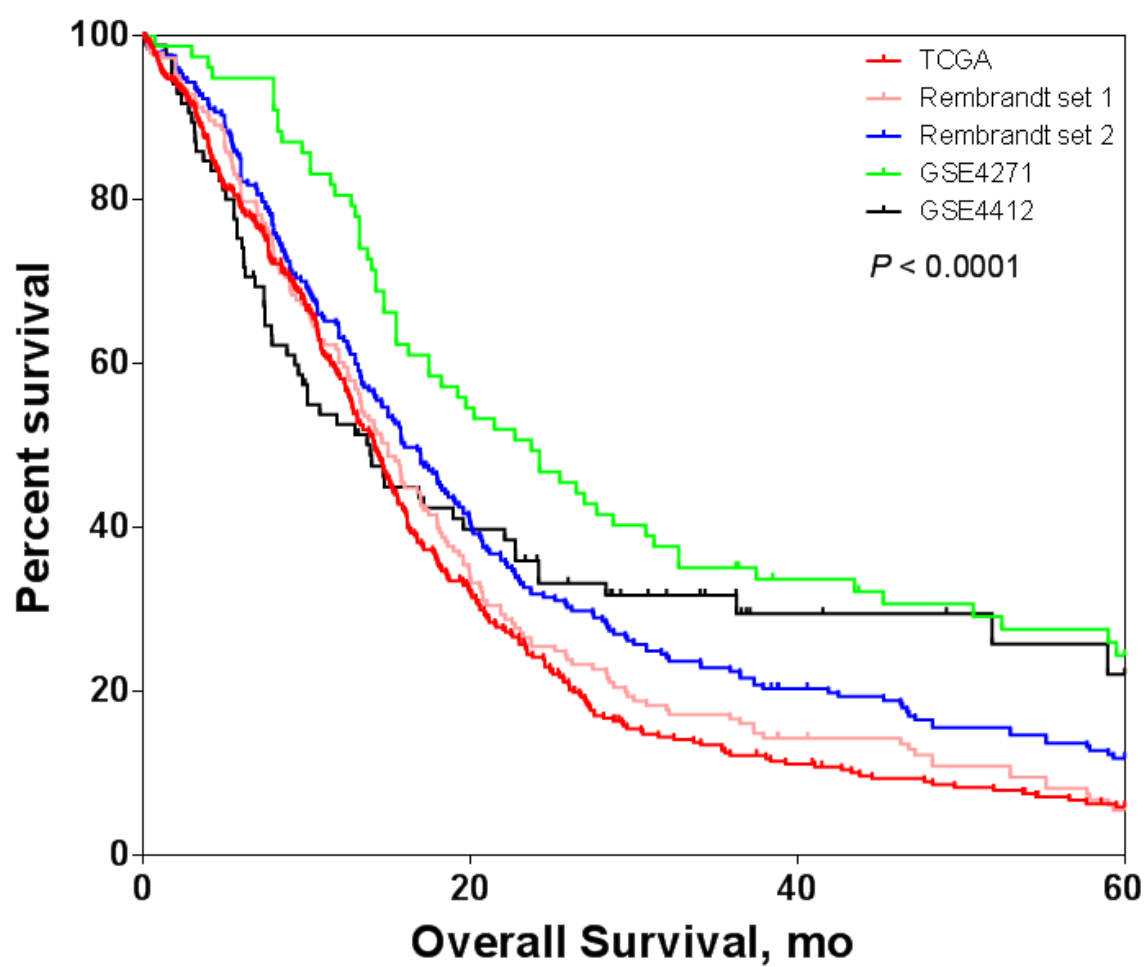

Supplement: Supplementary file 2 — Figure S2. Survival difference among the data sets that are included in this study. (PDF 57 kb) [file 12885_2018_4680_MOESM2_ESM.pdf]

Figure S3

(a) GSE4271

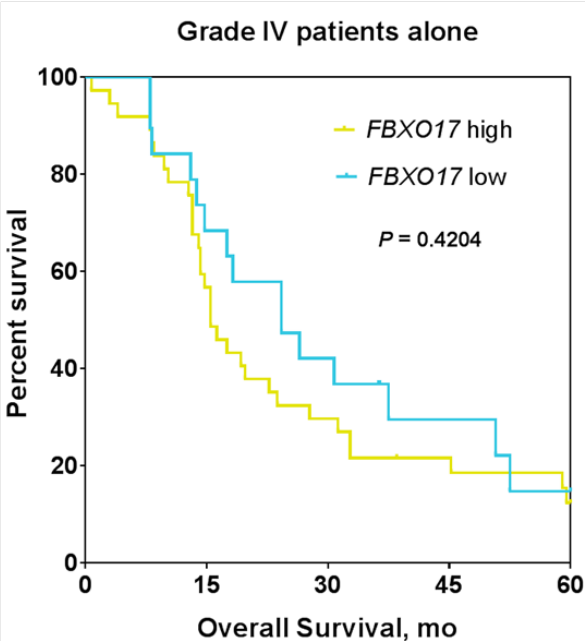

(b) GSE4412

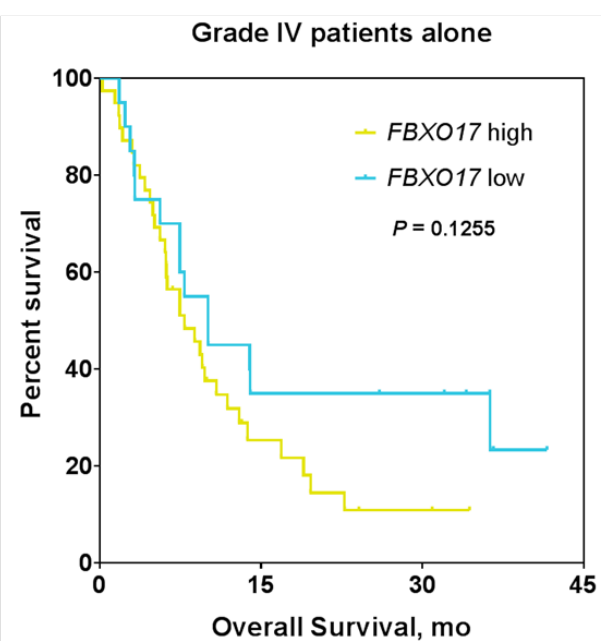

Supplement: Supplementary file 4 — Figure S3. Correlation of FBXO17 mRNA expression with survival within grade IV glioma patients alone, respectively, (a) in GSE4271 and (b) in GSE4412. The one-third patients with the least FBXO17 expression were categorized into FBXO17 low group and the rest of patients were categorized into FBXO17 high group. (PDF 113 kb) [file 12885_2018_4680_MOESM4_ESM.pdf]

Figure S4

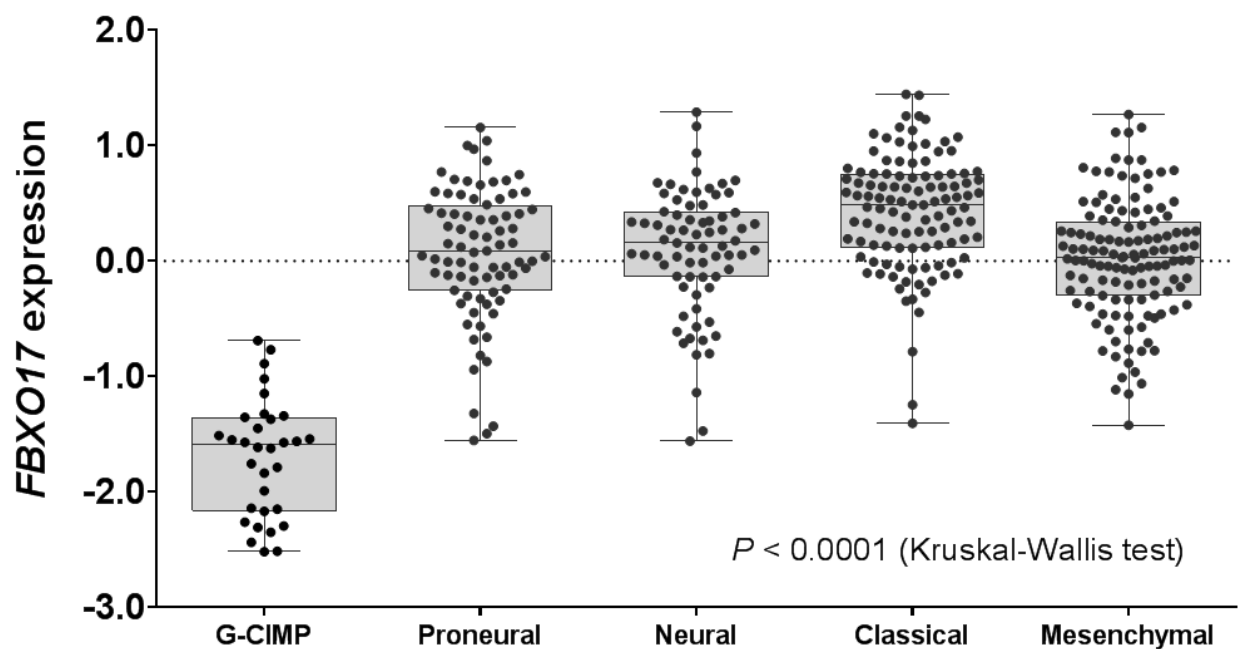

Supplement: Supplementary file 6 — Figure S4. Correlation of FBXO17 mRNA expression with GBM transcriptional subtypes in the TCGA cohort. (PDF 59 kb) [file 12885_2018_4680_MOESM6_ESM.pdf]

Figure S5

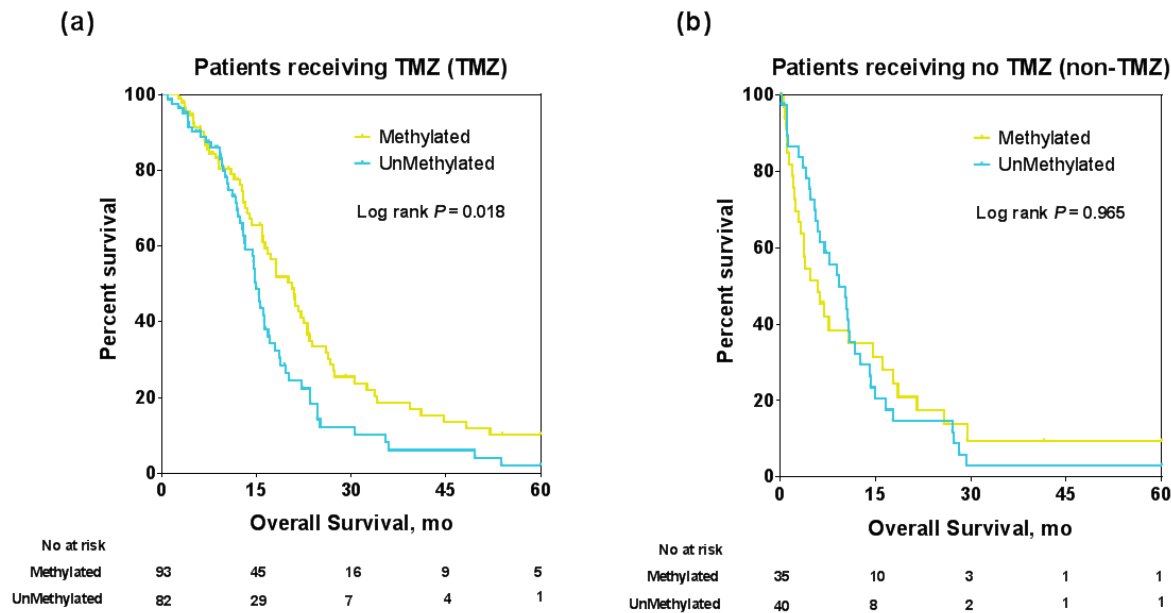

Supplement: Supplementary file 7 — Figure S5. (a) Kaplan-Meier OS curves for patients in the TMZ group after classifying patients into two groups based on MGMT status. (b) Kaplan-Meier OS curves for patients in the non-TMZ group after classifying patients into two groups based on MGMT status. (PDF 46 kb) [file 12885_2018_4680_MOESM7_ESM.pdf]

Figure S6

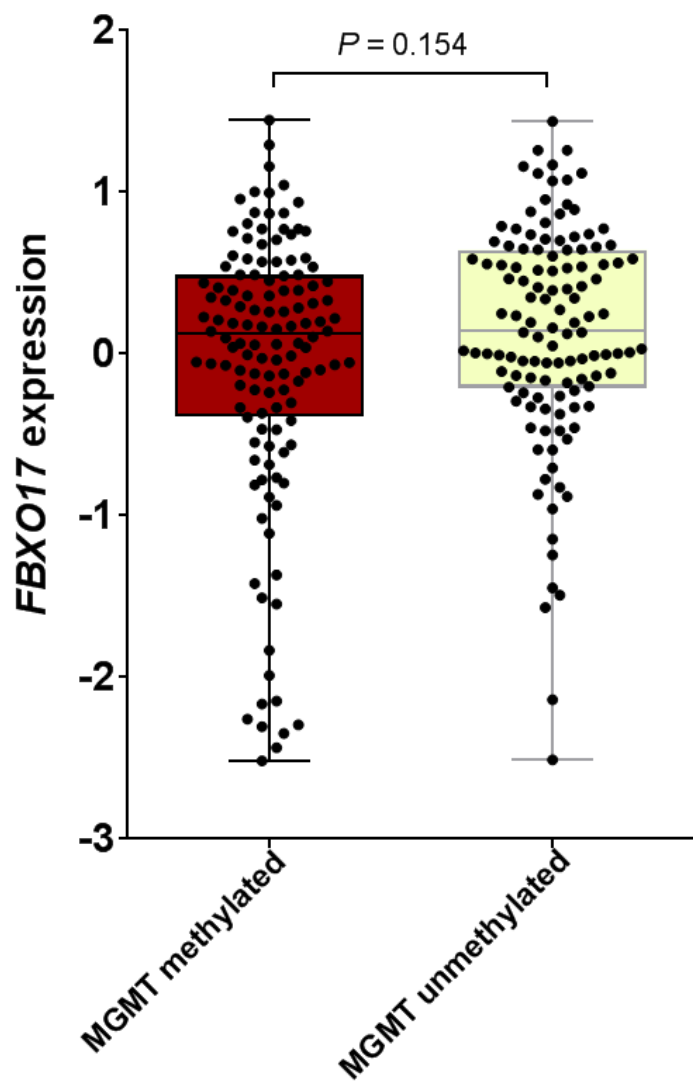

Supplement: Supplementary file 8 — Figure S6. Correlation of FBXO17 gene expression with MGMT methylation status in the TCGA cohort. The statistical significance was assessed by Mann-Whitney test. (PDF 65 kb) [file 12885_2018_4680_MOESM8_ESM.pdf]

Figure S7

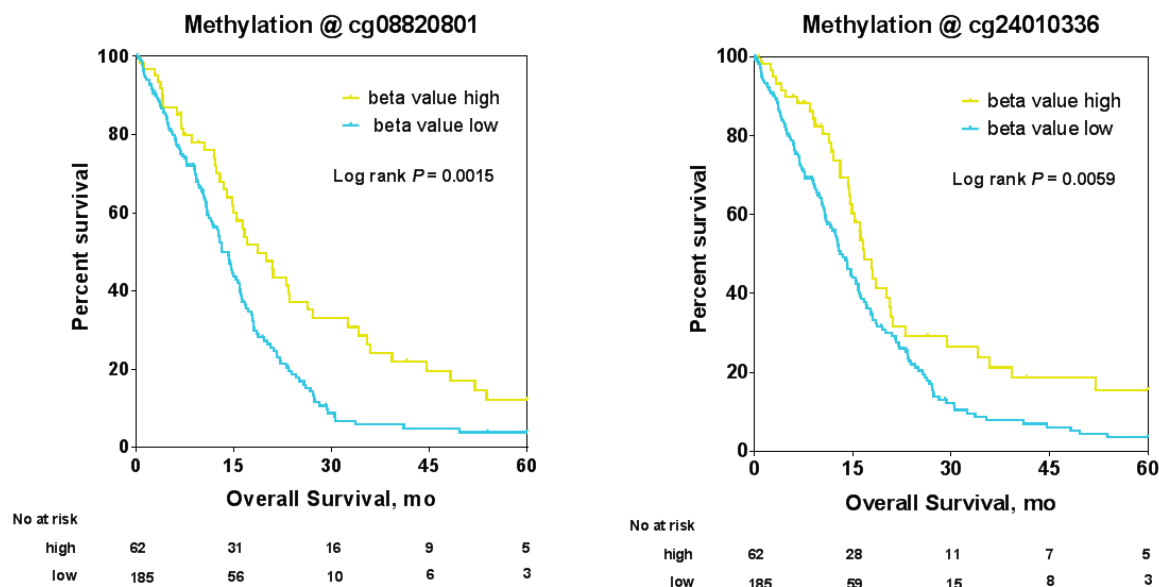

Supplement: Supplementary file 9 — Figure S7. Correlation of FBXO17 promoter methylation with patient survival in the TCGA cohort. (PDF 49 kb) [file 12885_2018_4680_MOESM9_ESM.pdf]
