# Supplementary material for: Clinical significance of FBXO17 gene expression in high-grade glioma
Source: BMC Cancer. 2018 Jul 31;18:773. doi: 10.1186/s12885-018-4680-3 (PMC6069786; doi:10.1186/s12885-018-4680-3)
Supplement: Supplementary file 3 — Table S1. The top 50 most significantly correlated genes with survival in the TCGA cohort. (DOCX 25 kb) [file 12885_2018_4680_MOESM3_ESM.docx]

**Table S1.** The top 50 most significantly correlated genes with survival in the TCGA cohort.

| Rank # | Gene | Cox coeff | *P* value | Rank # | Gene | Cox coeff | *P* value |
| --- | --- | --- | --- | --- | --- | --- | --- |
| 1 | RANBP17 | -0.3073 | 5.30E-09 | 26 | EFEMP2 | 0.2737 | 2.44E-05 |
| 2 | DIRAS3 | 0.2211 | 7.70E-07 | 27 | DYNLT3 | 0.2504 | 2.51E-05 |
| 3 | CLEC5A | 0.2678 | 1.02E-06 | 28 | C7orf13 | -0.1872 | 2.77E-05 |
| 4 | FBXO17 | 0.3394 | 2.14E-06 | 29 | CSTF2T | -0.3413 | 2.83E-05 |
| 5 | NSUN5 | 0.6385 | 3.36E-06 | 30 | ARL9 | 0.1785 | 3.03E-05 |
| 6 | RBM17 | -0.5012 | 3.38E-06 | 31 | FAM46A | 0.2660 | 3.63E-05 |
| 7 | EPC1 | -0.4611 | 3.46E-06 | 32 | BMP2 | -0.2096 | 3.77E-05 |
| 8 | LGALS8 | 0.3178 | 3.91E-06 | 33 | HVCN1 | 0.2754 | 4.01E-05 |
| 9 | KIAA0495 | 0.3447 | 6.04E-06 | 34 | CMYA5 | 0.1925 | 4.03E-05 |
| 10 | C9orf64 | 0.3950 | 7.23E-06 | 35 | HDHD3 | 0.4301 | 4.23E-05 |
| 11 | FAM60A | -0.4169 | 7.32E-06 | 36 | NUDT5 | -0.4477 | 4.54E-05 |
| 12 | CBLN1 | -0.2333 | 7.64E-06 | 37 | LGALS3 | 0.1291 | 4.59E-05 |
| 13 | H2AFY2 | -0.2356 | 8.33E-06 | 38 | MTHFS | 0.3994 | 4.60E-05 |
| 14 | LAMB4 | 0.2922 | 9.02E-06 | 39 | PRTFDC1 | -0.3062 | 4.63E-05 |
| 15 | MDK | 0.2884 | 1.07E-05 | 40 | CHL1 | 0.1578 | 4.64E-05 |
| 16 | FLJ32549 | 0.2995 | 1.17E-05 | 41 | GPRASP1 | 0.2461 | 4.72E-05 |
| 17 | SLC25A20 | 0.3207 | 1.27E-05 | 42 | IDS | 0.3140 | 4.82E-05 |
| 18 | FUCA2 | 0.3865 | 1.29E-05 | 43 | RARRES2 | 0.1296 | 4.85E-05 |
| 19 | EMP3 | 0.2598 | 1.39E-05 | 44 | CLIC1 | 0.2910 | 5.03E-05 |
| 20 | PGCP | 0.3153 | 1.43E-05 | 45 | SLC43A3 | 0.3835 | 5.17E-05 |
| 21 | NFKBIZ | 0.1971 | 1.53E-05 | 46 | WAC | -0.5240 | 5.19E-05 |
| 22 | IZUMO1 | -0.4828 | 1.68E-05 | 47 | GDI2 | -0.4373 | 5.25E-05 |
| 23 | SEPHS1 | -0.4452 | 1.76E-05 | 48 | EFNB2 | 0.3010 | 5.26E-05 |
| 24 | VASN | 0.1634 | 2.19E-05 | 49 | DKK3 | 0.3267 | 5.34E-05 |
| 25 | DCTD | 0.3872 | 2.27E-05 | 50 | FKBP9 | 0.2966 | 5.66E-05 |
