# Supplementary material for: Clinical significance of FBXO17 gene expression in high-grade glioma
Source: BMC Cancer. 2018 Jul 31;18:773. doi: 10.1186/s12885-018-4680-3 (PMC6069786; doi:10.1186/s12885-018-4680-3)
Supplement: Supplementary file 5 — Table S2. Univariate and Multivariate Models for Overall Survival in Patients with GBM in the TCGA Cohort while including MGMT methylation status as a covariate*. (DOCX 24 kb) [file 12885_2018_4680_MOESM5_ESM.docx]

**Table S2.** Univariate and Multivariate Models for Overall Survival in Patients with GBM in the TCGA Cohort while including *MGMT* methylation status as a covariate*.

|  | **Univariate analysis** | |  | **Multivariate analysis^‡^** | |
| --- | --- | --- | --- | --- | --- |
|  |  |  |  |  |  |
| **Overall Survival** | HR (95%CI)^†^ | *P* Value^§^ |  | HR (95%CI)^†^ | *P* Value^§^ |
|  | | | | | |
| *FBXO17* expression | 1.43 (1.17 –1.74) | **0.0004** |  | 1.17 (0.88 --1.55) | 0.274 |
| *IDH1* status |  |  |  |  |  |
| Wild-type (190) | 1 [Reference] |  |  | 1 [Reference] |  |
| Mutation (20) | 0.35 (0.18 – 0.67) | **0.001** |  | 0.50 (0.21 --1.21) | 0.126 |
| MGMT status |  |  |  |  |  |
| Unmethylated (105) | 1 [Reference] |  |  | 1 [Reference] |  |
| Methylated (105) | 0.73 (0.53 – 1.01) | 0.054 |  | 0.86 (0.62 --1.20) | 0.370 |
| Sex |  |  |  |  |  |
| Female (83) | 1 [Reference] |  |  | 1 [Reference] |  |
| Male (127) | 1.46 (1.05 – 2.03) | **0.026** |  | 1.60 (1.14 –2.26) | **0.007** |
| Age, years |  |  |  |  |  |
| < 60 (108)^ǁ^ | 1[Reference] |  |  | 1 [Reference] |  |
| ≥ 60 (102) | 2.02 (1.45 -- 2.80) | **<0.0001** |  | 1.77 (1.27 -- 2.48) | **0.001** |
| * Included are data from the 210 TCGA GBM patients who had all the following data available such as FBXO17 gene expression, IDH1 mutation, MGMT methylation status, gender, age, survival time, and vital status.  † HR, hazard ratio; CI, confidence interval.  ‡ Based on a multivariate Cox proportional hazards model, including all variables in the table.  § Wald’s test, *P* values.  ǁ The median age of the common set is 60 years. | | | | | |
